# How Self-Construals Affect Responses to Anthropomorphic Brands, With a Focus on the Three-Factor Relationship Between the Brand, the Gift-Giver and the Recipient

**DOI:** 10.3389/fpsyg.2018.02070

**Published:** 2018-11-05

**Authors:** Chien-Huang Lin, Yidan Huang

**Affiliations:** Department of Business Administration, National Central University, Taoyuan, Taiwan

**Keywords:** self-construal, anthropomorphic brands, top–down relationship, consumer–brand relationship, superior role, master, mentor

## Abstract

The universal mantra, “The customer is our king,” has led to considerable focus on the servant-anthropomorphized brand. However, does your “king” want to be served as a “king”? This research aims to examine how anthropomorphic brand role, self-construals and consumer responses to brands interact. In this study, four sequential experiments show that consumers with an interdependent self-construal are likely to respond more favorably toward anthropomorphic brands playing superior ‘master’ roles than toward those playing subordinate ‘servant’ roles. Here we distinguish between two types of superior role (master and mentor) based on behavior and communications. We also explore the underlying psychological mechanism of followership, as demonstrated through blind followership of someone in a master role and rational followership of someone in a mentor role. Additionally, when a third-party (recipient) is involved in the relationship between a consumer and a brand, the giver–recipient relationship moderates the relationship between an anthropomorphised brand role and self-construals.

## Introduction

Marketers often infuse brands with vivid human characters to increase the amount of attention they attract and to make them memorable (Aggarwal and McGill, 2007; Epley et al., 2007). State Farm declared itself a ‘good neighbor’ to its consumers. Consumers’ interactions with brands, even when these are one-way, assist in their view of the brand. This is especially pertinent nowadays with the role of social media in marketing and branding. Customers are now able to control brands in ways they were not able to until recently, and this has meant that marketers have needed to devise new means of interacting: one way is to seek to humanize the brand.

The literature largely focuses on the well-established effect of the role of ‘partner’ and ‘servant’ in brand anthropomorphism (Fournier, 1998; Fournier and Alvarez, 2012; Kim and Kramer, 2015; Gretry et al., 2017). Marketers try to make their consumers feel important and as if they have control over a brand. Being treated as a ‘king’ can strengthen a consumer’s attachment to a brand (Kim and Kramer, 2015). Additionally, securely attached individuals who view partners brand that can co-create value and work with them as basically supportive and trustworthiness (Paulssen and Fournier, 2007). However, brands play other social roles which do not fall into this taxonomy. Prior studies have merely covered bottom-up orientation (the servant role) or equality (the partner role) in the relationship between anthropomorphic brands and consumers. In this study, we are interested in top-down oriented relationships (superior roles). Thus, we explore how superiority works in marketing, in relationships between brands and consumers.

Although superior roles are universal in our daily lives, there are different types of superiority in human relationships. For example, parents, whose children need them for survival until they have become independent and who have the ultimate influence on their children’s lives, guide them to grow and develop in a different way to teachers; while teachers behave and work in a different way to employers. Thus, this study discusses two types of superior trait (master and mentor) in branding, based on how they express behavior and communicate with those they lead. We classify master and mentor as superior because they both possess the power to guide or schedule followers’ behaviors (Hartmann and Slapnièar, 2009). However, their downward-influence strategies are different. Masters tend to be powerful and charismatic superiors (Conger et al., 2000). For example, Hitler succeeded in establishing himself as a charismatic superior in the Nazi Party and required his followers to be unswervingly loyal; these followers, many of whom also occupied positions of superiority over others, remained bound to his authority until his death (Lepsius, 2006). Mentors tend to be dynamic and supportive superiors (Fagenson, 1989). Tony Dungy, the first black head coach to win the Super Bowl, stated that his focus on others was the secret to his own success (Pierce, 2011). Thus, those who follow masters are likely to be motivated by emotional attachment and irrational favors (Weber, 1968; Howell and Shamir, 2005) while those with mentors usually have reliable support that aids their development (Hunt and Michael, 1983; Byrne and Keefe, 2002). These different underlying psychological constructs are also found in consumer–brand relationships.

Undeniably, the corporate cliché of ‘the customer is king’ and the strategic concept of positioning brands as servants are popular marketing devices. However, it is also true that not everyone wishes to lead nor to feel in control all of the time. Love for a brand has been considered by psychologists studying individuals (Gómez-Suárez et al., 2017). Bornstein (1992) showed that individuals with an interdependent self-construal, who tended to follow instead of leading, performed better when working with those they felt to be superior to themselves rather than for those on a similar level (Earley, 1989; Singelis, 1994; Jung and Avolio, 1999). Thus, it is necessary to consider such self-construals in branding.

Additionally, previous studies on anthropomorphism in branding have only considered bilateral relationships between brands and consumers (Aaker, 1997; Aggarwal and McGill, 2007; Donnolley, 2012; Kim and Kramer, 2015). However, as the intent of a purchase from a brand is often to give to another person, bilateral relationships (givers and recipients) are transformed into triangular (or three-factor) relationships (brand, giver, recipient). Sherry (1983) suggests that gift-giving involves a social relationship between the giver and the recipient. Thus, we propose that the giver–recipient relationship also has an effect on anthropomorphic branding.

In this research, we argue that the role that anthropomorphic brands play interacts with self-construals to affect consumer attitudes toward brands. More specifically, our research has three goals: (1) to investigate the interactive effects of different anthropomorphic roles and consumer self-construals; (2) to explore the underlying psychological mechanisms of followership in contingent anthropomorphised roles and (3) to examine this interactive effect in different giver–recipient relationships.

## Theoretical Framework

### Anthropomorphic Brand Roles and Self-Brand Connections

Anthropomorphism can be defined as the attribution of uniquely human traits and characteristics to non-human objects or creatures (Epley et al., 2007). It is often used in marketing to create and enhance consumers’ feelings of connection to a brand.

Kim and Kramer (2015) demonstrated that differences in anthropomorphized brand roles imply different types of relationships, influencing consumer responses. It has been found that people relate to brands in a similar manner to how they relate to people (Kervyn et al., 2012), and thus marketers play on this to create the subliminal concept that brands are people. Anthropomorphism can thus help people to feel closer to a brand and to relate to it; this can result in brand love. Prior research on anthropomorphism in branding has identified two important and distinct roles which brands adopt. One is the partner role, whereby brands and consumers offer each other mutual benefits (Fournier, 1998; Fournier and Alvarez, 2012). The other is the servant role, whereby brands work to create benefits for consumers (Aggarwal and McGill, 2007; Kim and Kramer, 2015). However, little is known about the influence of top–down oriented relationships: i.e., the brand as superior to the consumer. As we have said, the superior role is universal in daily life. The democratic teacher plays a mentor role in students’ lives, and students are helped and encouraged to fulfill their potential (Sosik et al., 2004). A superior can be defined as the party in a relationship with the power to guide or schedule the other’s behavior (Hartmann and Slapnièar, 2009). However, due to variations in superiors’ behaviors and communication methods, the strategies they use to influence other parties differ (Manathunga, 2007; Carsten et al., 2010).

Both master leadership and mentor leadership follow the principles of social cognitive theory (Bandura, 1986), because master and mentor act as a role model who encourage development, and work to develop personal well-being (Sosik et al., 2004). And they both take downward influence strategies for follower, thus the mentor and master are classified as the leading roles in this paper. However, downward influence strategies have been divided into ‘hard,’ ‘rational’ and ‘soft’ behaviors (Kipnis and Schmidt, 1985; Yukl and Falbe, 1990). Combined with behaviors and communication methods of two roles, the master and mentor are conceptually distinct constructs (Scandura and Schriesheim, 1994; Egan, 2003). Hard strategies are often used by masters: the party in a relationship with the dominant status and the ability or power to control subordinates (Brief et al., 1991). Master leadership involves broadening and elevating followers’ goals and providing them with the confidence to go beyond minimally acceptable expectations of performance (Bass and Avolio, 1997). Personal charisma is especially important in charismatic leadership (Conger et al., 2000). Their tactics often involve using pressure (commands or threats) and coalition (using co-workers to create pressure to comply) to influence subordinates. In contrast, mentors are generally defined as influential senior or higher-ranking people who are committed to supporting their followers (Hunt and Michael, 1983). Mentoring leadership involves an individual with more advanced experience and knowledge (mentor) who assists a less-experienced and knowledgeable protégés with personal and professional development (Levinson, 1978). Mentors often use rational influencing strategies that include exchanges (offering practical benefits in return for followers’ engagement) and rational persuasion (using logical arguments, and professional experience and advanced knowledge).

### Self-Construals and Anthropomorphic Brand Roles

Even in a top–down style of leadership, both leaders and followers have important, active roles in a relationship. The social identity theory of leadership (Hogg, 2001) demonstrates that the effectiveness of a leader–member relationship is built on how members identify their relationships and how they form self-concepts within these relationships. Prior research has demonstrated that followers’ personal characteristics, emotions and attitudes influence their perceptions of or preferences for certain types of relationship with superiors and propensity to follow a particular type of leader (Ehrhart and Klein, 2001; Kark et al., 2003).

The social cognition theory has developed a sustainable theoretical construct to demonstrate self-construal and self-related behaviors. A self-construal can be defined as a collection of feelings, actions and thoughts concerning the self: how a person thinks about and defines themselves, and how they relate to the wider world (Triandis, 1989; Singelis, 1994). We investigate two self-construal aspects, interdependent and independent, in this article. An interdependent self-construal is conceptualized as someone perceiving themselves to have a variable, flexible self. Individuals with an interdependent self-construal believe they are intertwined with others and are impressionable: they can be molded in situations. In contrast, an independent self-construal is conceptualized as someone feeling they have a ‘unitary stable, bounded’ self. Individuals with an independent self-construal are relatively separate from their social context; they often express themselves directly and say directly what they think; they are unlikely to be heavily influenced by others’ feelings or actions and do not readily change their thinking (Markus and Kitayama, 1991; Singelis, 1994).

Interdependent people are expected to readily identify with their superiors’ goals or the common goals of the group. Bornstein (1992) demonstrated that interdependent people tend to follow others with alacrity and seek out dependent relationships with superiors. Conger et al. (2000) demonstrated that master leadership will be positively related to followers’ sense of collective identity. They perform well when working with superiors and present high levels of loyalty (Earley, 1989; Jung and Avolio, 1999). Independent people, in contrast, tend to be self-interested and place a high priority on personal goals when working in a team. They often welcome anyone who can help their need for personal development and are motivated by useful people in lesser roles to them but they generally dislike being told what to do (Hofstede, 1980; Jung and Avolio, 1999; Burroughs and Rindfleisch, 2002).

As such, we proposed the following hypotheses:

H1: Individuals with an interdependent self-construal will respond with more affection to superior (master and mentor) anthropomorphized brand roles.
H2: Individuals with an independent self-construal will respond with more affection to servant anthropomorphized brand roles.

### Blind Followership and Rational Followership

Authoritative people who lead via control and the issuing of orders, and who expect obedience, are defined here as ‘masters.’ These people seem like commanders when they convey an instruction (e.g., “you should obey me”) (Brief et al., 1991) and this can be attractive to people who like to be told what to do, often without really thinking about it too much: they are defined here as blind followers. Superiors who are domineering and yet charming are often described as exhibiting charismatic and transformational leadership styles. Masters display self-confidence that has an impact on their followers. They tend to provide a vision that can reduce uncertainty and fear by clearly defining situations (Hollander, 1992). Since this personalized relationship is formed by followers with interdependent self-construal or low self-concept clarity, and because such a relationship includes idealization and romanticization of the leader, followers who form this type of relationship are likely to be prone to “blind” faith in the leader and to “hypercompliance” (Zablocki, 1999) and unquestioning obedience to the leader. The blind follower is motivated by emotional attachment and often irrational favor (Weber, 1968; Howell and Shamir, 2005). Mao Tse-Tung, as a typical master, his followers are so crazy that take the book “Quotations from Chairman Mao” as wedding gifts during the Chinese Cultural Revolution (Schram, 1967; Pye, 1976; Carey, 1992; Cook, 2014). Respect for and increased conformity to masters increases blind followers’ confidence in their choices (House, 1977; Shamir et al., 1993; Blass, 1999).

Mentors play a more democratic and supportive leadership role than masters (Fagenson, 1989). With mentors’ guidance, advice and counsel, mentoring forms a voluntary alliance between leaders and their followers. Contrary to the blind obedience style of leadership practiced by masters, this is a more rational followership because mentors can provide reliable support to enable development (Hunt and Michael, 1983; Byrne and Keefe, 2002). Followers in mentoring relationship evaluate mentor self-presentations as a means for achieving their own goals. The decision to support a particular mentor depends on the goals of the followers, the information available about other mentor’ presentations, and the followers’ strategies for evaluating mentor and enforcing their choices. The process of deciding is one subject to rational calculation (Nakamura, 1980). With mentoring, protégés’ motivation and satisfaction tends to increase (Fagenson, 1989; Dreher and Ash, 1990). Students can achieve professional benefits from relationships with mentors (Hudson, 2013) and, in schools, mentor teachers use professional knowledge and skills to guide students and help them progress. Professional qualities, such as knowledge and experience, as well as self-confidence, are necessary to encourage people to become followers.

Those with interdependent self-constructs are likely to follow others and superiors (a master or a mentor) can increase their confidence. Thus, marketers can use anthropomorphized brand roles who behave in a superior manner, as if they were masters or mentors, to engage these people and to encourage the expression of a favorable response to the brand. Attitudinally, they like to feel subservient to a brand just as they like to feel subservient in a relationship to someone in authority, whether this is a master or a mentor. Those with independent self-constructs are unlikely to respond in such a way to a type of marketing that posits that a brand is their superior because they tend to prefer to be served and followed.

Thus, we proposed the following:

H3: Blind followership mediates the effects of interactions between the master anthropomorphism role and self-construal.
H4: Rational followership mediates the interaction effect of the mentor anthropomorphism role and self-construal.

### The Giver–Recipient Relationship With an Anthropomorphic Brand Roles

Extant research has verified the popularity of brand anthropomorphism, but most research into this only relates to the relationship between consumers and brands (Aaker, 1997; Aggarwal and McGill, 2007; Donnolley, 2012; Kim and Kramer, 2015). However, the majority of connections in life are multilateral: for example, a nuclear family comprising a couple and their children. There is a paucity of knowledge on the interactive relationships between consumers and brands when a third party enters the relationship, such as the recipient of a gift that will affect the choice of someone deciding on which brand to buy. Thus in this investigation into the influence of brand anthropomorphism and self-construals we add a third party: the gift recipient.

The gift-giving multidisciplinary model proposed by Sherry (1983) has three main components: gifts, the relationship between the gift-giver and the recipient, and the giving situation. Based on this model, the gift-giving process comprises three stages: gestation, prestation and reformulation. Specifically, gestation is similar to the traditional consumer decision-making model. During this stage, decision-making is influenced by the social relationship between givers and recipients and by givers’ surmises of recipients’ needs (Belk, 1977; Wagner et al., 1990). Givers make decisions based on their relationships with recipients (e.g., a gift to a best friend may cost a lot more than for one for a new neighbor) and the recipients’ personalities (a giver may choose a conservative gift for an introverted recipient).

Prior research has focused on such bi-lateral relationships between gift-givers and recipients (Belk, 1977; Sherry, 1983; Ward and Broniarczyk, 2011). Due to brand involvement, bilateral gift-giving relationships between givers and recipients are transformed into tri-partite (or three-factor) relationships: brands, givers and recipients. Consumers will take their relationship to a recipient into account as a buying factor when they choose a gift and also take into consideration the ‘personality’ of the brand. In terms of self-construals, interdependent people are more concerned with others’ needs than independent people: hence the giver–recipient relationship has a more significant influence on the former than the latter.

Thus, we proposed the following hypothesis:

H5: The giver–recipient relationship moderates the relationship between an anthropomorphic brand and self-construals.

Given gift selection is based on givers’ perceptions of recipients’ needs, when the recipients are subordinates a brand which uses anthropomorphism to create servile characters may well be used. However, when the recipients are superiors, the givers are likely to believe the recipients need subordinates to serve them, so superior (master and mentor) anthropomorphic characters are likely to be perceived as suiting the recipients’ needs. Due to the level of involvement and form of donor-recipient relationships (Wagner et al., 1990), we expected perceived relationship orientation to affect the interactive effect between anthropomorphic brand and self-construals. Thus, we proposed the following hypotheses:

H6: When gift-givers with an interdependent self-construal perceive recipients as subordinates in their relationships, they respond with more affection to a brand using master/mentor anthropomorphism.
H7: When gift-givers with an interdependent self-construal perceive recipients as superiors in their relationships, they respond with more affection to a brand using subordinate anthropomorphism.

## Experiment 1

### Participants

In the first experiment conducted for this research, 347 graduate students aged between 25 and 37 (*M* = 30.1, *SD* = 3.19, 48% female) from a variety of disciplines at a university in Taiwan participated in exchange for a meal ticket and a chance to enter a $50 lottery. The study was carried out in accordance with the recommendations of the National Central University Research Committee and with written informed consent from all of the participants, in accordance with the Declaration of Helsinki.

### Experimental Design and Procedure

The experiment involved two manipulated factors (anthropomorphism role: master or mentor vs. servant; self-construal: independent vs. interdependent). The participants were invited to evaluate a fictitious brand that produced household appliances. We did not provide the brand name to avoid the influence of subjective brand perception and chose a washing machine for the experimental target as it is a common appliance.

Consistent with prior research (Aggarwal and McGill, 2007; Kim and Kramer, 2015), the participants were presented with photos of anthropomorphic appliances, using a verbal cue to elicit their responses (see Appendix A). The participants in the master condition were exposed to the following slogan: “Obey me, give yourself a comfortable life!” In the mentor condition, the slogan was, “With my help, give yourself a comfortable life!” In the servant condition, the slogan was “Let me be your servant, give yourself a comfortable life!”

Next, we manipulated the self-construal prompts in a similar manner to Trafimow et al. (1991). In the independent prime, the participants were told to “Please take 5 min to think and write down how you are different from your family and friends.” In the interdependent prime, the participants were told to “Please take 5 min to think and write down how you are similar to your family and friends.”

Additionally, we expected emotional followership in a master role and rational followership in a mentor role to mediate the brand evaluation separately so used the phrases “I feel the confidence and charm of the brand, and I believe the choice is right” and “The brand can give me reliable and practical suggestions” in the testing.

We pre-tested the mediation valance in line with the experiment of Kim and Kramer (2015), with 69 (*M* = 31, *SD* = 1.79, 51% female) participants randomly assigned to two conditions (“I feel the confidence and charm of the brand, and I believe the choice is right” or “The brand can give me reliable and practical suggestions”). They were told to evaluate the type of follower who would concur with these statements (“I think the followers are emotional” and “I think the followers are rational,” anchored by 1 = strongly disagree and 7 = strongly agree). The results supported the idea that the manipulation was effective, as the “I feel the confidence and charm of the brand, and I believe the choice is right” situation resulted in stronger emotional perceptions (*M*emotional = 4.78 vs. *M*rational = 3.06, *SD* = 0.83 vs. 1.26, β = 1.37, *t* = 7.08, *P* < 0.01). The participants generally agreed that this sentence expressed an emotional type of followership, with the mean score for emotional followership being significantly higher than that for rational followership. Meanwhile, the statement “The brand can give me reliable and practical advice” resulted in strongly rational perceptions (*M*emotional = 3.32 vs. *M*rational = 4.64, *SD* = 1.05 vs. 0.98, β = 1.38, *t* = -5.86, *P* < 0.01).

### Measures

Firstly, we tested the manipulation (anthropomorphic branding and self-construal) valance. To test the role of anthropomorphism in manipulation, the participants’ reactions were assessed in terms of whether they considered the branding of the appliance to reflect mastery (“The brand looks like a master to me” and “I would obey the brand,” *r* = 0.81), mentoring (“The brand looks like a mentor to me” and “the brand would help me,” *r* = 0.88) and subservience (“The brand looks like a servant to me” and “The brand would obey me,” *r* = 0.88), anchored by 1 = strongly disagree and 7 = strongly agree. To test their self-construal, the participants were instructed to complete 10 statements beginning with “I am __.” after manipulation (Trafimow et al., 1991; Brewer and Gardner, 1996; Gardner et al., 1999).

The participants indicated their attitudes toward the advertised brand on a four-item, seven-point scale anchored by ‘bad/good,’ ‘unfavorable/favorable,’ ‘negative/positive’ and ‘unappealing/appealing’ (Jeong, 2008; Chang et al., 2017). We then assessed the reason for favoring a brand by asking the participants to rank “I feel the confidence and charm of the brand, and I believe the choice is right” and “The brand can give me reliable and practical advice,” anchored by 1 = strongly disagree and 7 = strongly agree. Lastly, the participants completed demographic questions: i.e., gender and age.

### Results

#### Manipulation Check

As expected, the master manipulation resulted in stronger master perceptions (*M*master = 5.07 vs. *M*mentor = 3.36, *M*servant = 3.26) and the mentor manipulation in stronger mentor perceptions (*M*mentor = 5.06 vs. *M*master = 3.30, *M*servant = 3.31). Additionally, the servant manipulation resulted in stronger servant perceptions (*M*servant = 5.02 vs. *M*master = 3.12, *M*mentor = 3.44).

We invited two independent researchers to code and judge self-construal statements as either interdependent or independent. Independent statements referred to a personal attitude, description, or belief (e.g., I am clever). Interdependent statements included either a category or group relative to belonging (e.g., I am a Buddhist) or a relationship to others (e.g., I am an older brother). Invalidity statements (e.g., I am tired of this survey) were excluded from the analysis. As a result, the participants in the independent prime were found to write more individualistic statements than those in the interdependent prime (*M* = 5.62 vs. 4.37, *SD* = 1.12 vs. 1.12, *t* = -6.43, *p* < 0.01), whereas participants in the interdependent prime wrote more collectivistic statements than those in the independent prime (*M* = 5.57 vs. 4.43, *SD* = 1.23 vs. 1.23, *t* = 6.18, *p* < 0.01).

#### Attitudes Toward the Advertised Brand

A 3 (anthropomorphized role: master, mentor and servant) ^∗^ 2 (self-construal: independent, interdependent) analysis of variance (ANOVA) test on brand attitudes indicated that the interaction of brand anthropomorphism with self-construals was significant [*F*(2,341) = 23.19, *p* < 0.01, η^2^ = 0.14) (see Table 1).

**Table 1:** Results of two-way analysis of variance for brand attitude.

	*SS*	*df*	*MS*	*F*	*p*	Cohen’ *f*	*Achieved power*
Anthropomorphism role (A)	4.02	2	2.01	1.70	0.18	0.11	0.36
Self-construal (B)	11.46	1	11.46	9.70	0.00	0.14	0.77
A^∗^B	54.77	2	27.39	23.19	0.00	0.35	0.98
Error	402.75	341	1.18				

*^∗^*p*-value ≤ 0.05; ^∗∗^*p*-value ≤ 0.01; and ^∗∗∗^*p*-value ≤ 0.001.*

**FIGURE 1:**
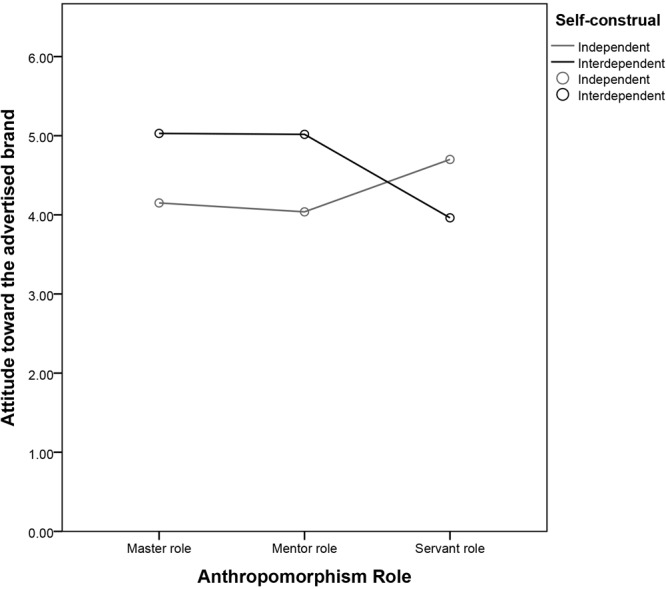
Attitude toward the advertised brand as a function of the anthropomorphism role and self-construal.

We then undertook a post-hoc analysis. In the master role condition, interdependent consumers responded with more affection than independent consumers [*M*ind = 4.15 vs. *M*int = 5.03, *SD* = 1.01 vs. 1.39; *F*(1,341) = 16.63, *P* < 0.01]. In addition, interdependent consumers responded with more affection than independent consumers in the mentor role condition [*M*ind = 4.04 vs. *M*int = 5.02, *SD* = 1.03 vs. 0.86; *F*(1,341) = 22.67, *P* < 0.01]. Independent consumers responded with more affection than interdependent consumers in the servant role condition [*M*ind = 4.71 vs. *M*int = 3.96, *SD* = 1.11 vs. 1.18; *F*(1,341) = 14.39, *P* < 0.01] (see Figure 1 and Table 2). This result supports H1 and H2.

**Table 2:** Post-test between Anthropomorphism role (AR) and Brand Attitude (BA).

AR\BA	Mean and SD	*F*	*P*
Master role frame	*M*ind = 4.15, *SD* = 1.01 vs. *M*int = 5.03, *SD* = 1.39	16.63	^∗∗∗^
Mentor role frame	*M*ind = 4.04, *SD* = 1.03 vs. *M*int = 5.02, *SD* = 0.86	22.67	^∗∗∗^
Servant role frame	*M*ind = 4.71, *SD* = 1.11 vs. *M*int = 3.96, *SD* = 1.18	14.39	^∗∗∗^

*^∗^*p*-value ≤ 0.05; ^∗∗^*p*-value ≤ 0.01; and ^∗∗∗^*p*-value ≤ 0.001.*

#### Mediation Analysis

A regression analysis was performed on consumers’ attitude for brand using the following independent variables: self-construals (interdependent = 1, independent = -1) and two dummy variables for the three priming manipulations: dummy1 variable for the mentor role condition (coded as one for mentor role and zero for master role and servant role) and a dummy2 variable for the master role condition(coded as one for the master role priming and zero for mentor role and servant role). We employed Model 8 from Hayes (2018) with 5000 resamples to test moderated mediation effect.

The dummy1 × self-construal interaction predicted blind followership (*b* = 1.48, *t* = 4.20, *p* < 0.05). Also, the dummy2 × self-construal interaction predicted blind followership (*b* = 2.43, *t* = 8.20, *p* < 0.01). Second, the model regressed blind followership on brand attitude, the brand role, self-constural, and the interaction of the last two factors. Blind followership predicted brand attitude (*b* = 0.15, *t* = 2.91, *p* < 0.01). Additionally, the dummy1 × self-construal interaction predicted rational followership (*b* = 2.70, *t* = 10.50, *p* < 0.01). The dummy2 × self-construal interaction predicted rational followership (*b* = 0.66, *t* = 2.50, *p* = 0.01). And the model regressed rational followership on brand attitude, the brand role, self-constural, and the interaction of the last two factors. Rational followership predicted brand attitude (*b* = 0.16, *t* = 2.72, *p* < 0.01).

Most importantly, bootstrapping analysis revealed that blind followership mediated the interactive effect of self-construal and dummy2 on brand attitude in interdependent-self condition (95% CI: 0.10 – 0.72). And rational followership mediated the interactive effect of self-construal and dummy1 on brand attitude in interdependent-self condition (95% CI: 0.06 – 0.82). However, in dependent-self condition, the blind followership did not mediated the interactive effect of self-construal and dummy2 (95% CI: -0.04 – 0.11), while the rational followership did not mediated the interactive effect of self-construal and dummy1 (95% CI: -0.06 – 0.07). Taken together, the result suggests that blind (rational) followership mediates the effects of interactions between the master (mentor) anthropomorphism role and self-construal, which support H3 and H4.

## Experiment 2

### Participants

Three hundred and eighty-two (*M*age = 29.5, *SD* = 2.51, 58% female) graduate students participated in this study in exchange for a meal ticket and entrance into a $50 lottery. All subjects provided written informed consent in accordance with the Declaration of Helsinki, as for Experiment 1.

### Experimental Design and Procedure

The experiment involved three manipulated factors (anthropomorphism role: master or mentor vs. servant; relationship orientation between giver and recipient: top–down vs. down–top; self-construal: independent vs. interdependent). The participants were asked to evaluate a brand of phone. They were presented with a photo of the phone with the strapline: “A phone brand is going to launch a new phone. Please imagine you want to buy a phone of this brand as a gift for your friend.”

We next manipulated the anthropomorphism roles and self-construals similar to in Experiment 1. There were changes made to the advertisements’ slogans due to the change to the target product. In the master condition, the participants received the following slogan: “Obey me, give yourself a smart life.” In the mentor condition, the slogan was “With my help, give yourself a smart life.” In the servant condition, the advertisement slogan was “Let me be your steward, give yourself a smart life” (see Appendix B). In the top-down relationship condition, the participants read “The gift is for the friend who often depends on or need you.” In the down-top relationship condition, participants read “The gift is for the friend whom I follow and depend on.”

### Measures

Firstly, we tested the manipulation (type of anthropomorphism, self-construal) valance, as in Experiment 1. Next, the participants measured top–down perceptions via the statements “You and the recipient have a top–down relationship” and “You and the recipient relationship have a bottom–up relationship,” anchored by 1 = strongly disagree and 7 = strongly agree. The participants then indicated their attitude toward the advertised brand on a four-item, seven-point scale anchored by ‘bad/good,’ ‘unfavorable/favorable,’ ‘negative/positive,’ and ‘unappealing/appealing’ (Jeong, 2008; Chang et al., 2017). Finally, the participants completed demographic questions: i.e., gender and age.

### Results

#### Manipulation Check

As expected, the master manipulation resulted in stronger master perceptions (*M*master = 5.16 vs. *M*mentor = 3.67, *M*servant = 3.37), and the mentor manipulation resulted in stronger mentor perceptions (*M*mentor = 5.36 vs. *M*master = 3.31, *M*servant = 3.47). Additionally, the servant manipulation resulted in stronger servant perceptions (*M*servant = 5.63 vs. *M*master = 3.88, *M*mentor = 3.63).

We invited two independent researchers to code and judge each statement as either interdependent or independent, as for Experiment 1. As a result, the participants in the independent prime wrote more individualistic statements than those in the interdependent prime (*M* = 5.88 vs. 4.12, *SD* = 1.09 vs. 1.09, β = 2.19, *t* = 11.26, *p* < 0.01), whereas the participants in the interdependent prime wrote more collectivistic statements than those in the independent prime (*M*colle = 5.79 vs. *M*indiv = 4.21, *SD* = 1.26 vs. 1.26, β = 2.53, *t* = -8.54, *p* < 0.01).

Additionally, the top–down relationship manipulation resulted in stronger top–down perceptions (*M*top–down = 4.65 vs. *M*down–top = 4.22, *SD* = 1.27 vs. 1.49, *t* = 2.29, *p* < 0.01), and the down–top relationship manipulation resulted in stronger down–top perceptions (*M*top–down = 4.37 vs. *M*down–top = 4.89, *SD* = 1.53 vs. 1.41, *t* = 2.48, *p* < 0.01).

#### Attitudes Toward the Advertised Brand

A 3 (anthropomorphized role: master, mentor, servant) ^∗^2 (self-construal: independent, interdependent) ^∗^(relationship orientation between givers and recipients: top–down vs. down–top) ANOVA test on brand attitudes did not reveal a primary three-way effect of anthropomorphized roles [*F*(2,370) = 0.20, *p* = 0.89, η^2^ = 0.001] but was qualified by the predicted interaction between relationship orientations and anthropomorphized roles [*F*(2,370) = 20.03, *p* < 0.01, η^2^ = 0.12]. However, no interaction was found between anthropomorphized roles and self-construals [*F*(2,370) = 0.86, *p* = 0.42, η^2^ = 0.001] (see Table 3). The result supports H5.

**Table 3:** Results of three-way analysis of variance for brand attitude.

	*SS*	*df*	*MS*	*F*	*p*	Cohen’ *f*	*Achieved power*
Anthropomorphism role (A)	0.62	2	0.31	0.35	0.71	0.04	0.11
Self-construal (B)	0.03	1	0.03	0.30	0.86	0.03	0.05
Relationship orientation (C)	1.29	1	1.30	1.44	0.23	0.05	0.18
A^∗^B	1.56	2	0.78	0.86	0.42	0.06	0.18
A^∗^C	36.15	2	18.07	20.03	0.00	0.32	0.99
B^∗^C	1.85	1	1.85	2.05	0.15	0.07	0.22
A^∗^B^∗^C	0.20	2	0.10	0.11	0.89	0.03	0.07
Error	333.79	370					

*^∗^*p*-value ≤ 0.05; ^∗∗^*p*-value ≤ 0.01; and ^∗∗∗^*p*-value ≤ 0.001.*

**FIGURE 2:**
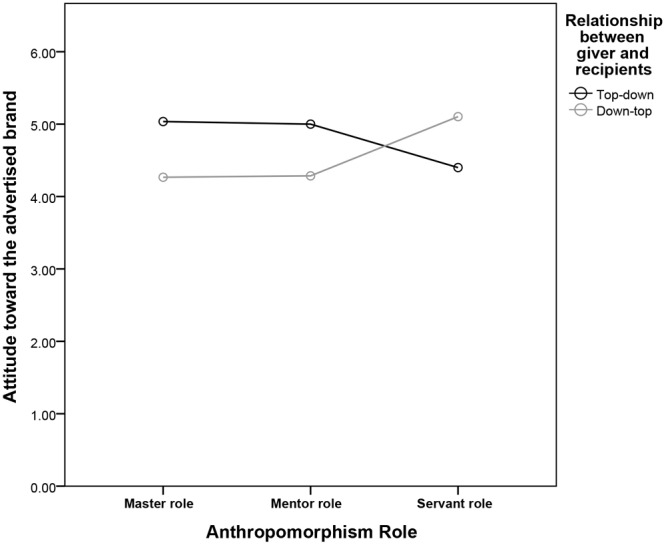
Interdependent gift-giver’s attitude toward the advertised brand as a function of anthropomorphism role and relationship between giver and recipients.

We then undertook a *post hoc* analysis for interdependent gift-giver. In the master role condition, the top–down givers responded with more affection than the down–top givers consumers [*M*top–down = 5.03 vs. *M*down–top = 4.27, *SD* = 0.74 vs. 0.98; *F*(1,376) = 9.04, *P* < 0.01]. In addition, the top–down givers responded with more affection than the down–top givers consumers in mentor role condition [*M*top–down = 5.00 vs. *M*down–top = 4.29, *SD* = 1.01 vs. 1.06; *F*(1,376) = 11.35, *P* < 0.01]. However, the down–top givers responded with more affection than the top–down givers consumers in the servant role condition [*M*top–down = 4.40 vs. *M*down–top = 5.10, *SD* = 0.56 vs. 1.26; *F*(1,376) = 7.70, *P* < 0.01] (see Figure 2 and Table 4). The result supports H6 and H7.

**Table 4:** Post-test between Anthropomorphism role (AR) and Brand Attitude (BA).

AR\BA	Independent gift-giver			Interdependent gift-giver		
	Mean and SD	*F*	*P*	Mean and SD	*F*	*P*
Master role frame	Mtop-down = 4.78, *SD* = 0.91 vs. Mdown-top = 4.40, *SD* = 0.95	2.40	0.12	Mtop-down = 5.03, *SD* = 0.74 vs. Mdown-top = 4.27, *SD* = 0.98	9.04	^∗∗∗^
Mentor role frame	Mtop-down = 5.01, *SD* = 1.03 vs. Mdown-top = 4.59, *SD* = 0.79	3.12	0.08	Mtop-down = 5.00, *SD* = 1.01 vs. Mdown-top = 4.29, *SD* = 1.06	11.35	^∗∗∗^
Servant role frame	Mtop-down = 4.17, *SD* = 0.62 vs. Mdown-top = 4.47, *SD* = 1.04	2.89	0.22	Mtop-down = 4.40, *SD* = 0.56 vs. Mdown-top = 5.10, *SD* = 1.26	7.70	^∗∗∗^

*^∗^*p*-value ≤ 0.05; ^∗∗^*p*-value ≤ 0.01; and ^∗∗∗^*p*-value ≤ 0.001.*

## Experiment 3

This experiment serves for multiple purposes. First, we aim to test if our proposing effect is influenced by target product selection. Although we choose goods at random, they both belong to high-involvement product. The high-involvement product represents the consumer’s personality, status and lifestyle. These products are usually expensive and require consumers to spend more time to make purchase decisions, for example, buying a home theater. By contrast, low- involvement products are those that reflect routine purchase decisions; for example, buying a candy or an ice cream. And previous research found that brand loyalty would interact with product involvement (Park, 1996; Leclerc and Little, 1997). Thus, we use a opposite product (low-involvement) in Experiment 3 to test whether product choice would make influences on interactive effect between anthropomorphic brands and self-construal. Second, in order to eliminate the interference of experiment process to the result, we seek to replicate the previous result by adjusting the process of experiment. In Experiment 3, we manipulate the self-construal first and then are followed by brand anthropomorphic manipulation.

### Participants

A total of 251 (*M*age = 27.5, *SD* = 2.35, 44% female) graduate students participated in this study in exchange for a lunch ticket and entrance into a $50 lottery. All subjects provided written informed consent in accordance with the Declaration of Helsinki, similar to study 1 and 2.

### Experimental Design and Procedure

The experiment involved two manipulated factors (anthropomorphism role: master or mentor vs. servant; self-construal: independent vs. interdependent). First, we manipulated the self-construal prompt similar to that of Trafimow et al. (1991). In the independent prime, the participants were told to “Please take 5 min to think and write down how you are different from your family and friends.” In the interdependent prime, the participants were told to “Please take 5 min to think and write down how you are similar to your family and friends.”

Next, we manipulated the anthropomorphism role. The participants were invited to look through an advertisement photo (Appendix C) and evaluate a fictitious FMCG brand that want to launch a new toothpaste product. We chose a toothpaste as our experimental target because it is a common family product and it belong to low-involvement product with lower price.

Participants in the Experiments 1 and 2 both received the pre-defined anthropomorphic verbal cue made by research and measure their superior and servant role perceptions. Besides, the similar slogans limit the generalizability. In order to addresses this limitations, we use the different manipulated role method in Experiment 3. The participants in the master condition were exposed to the following instruction: “The brand wants to present its as a master to the customer. Briefly describe how it can make the consumer follow and obey it.” Those in the mentor condition read, “The brand wants to present its as the one that mentor with the customer. Briefly describe how it can guide and support the consumer.” Those in the servant condition read, “The brand wants to present its as one that serves the customer. Briefly describe how it can serve and work for you.” The participants were asked to write down their ideas.

We had pre-tested the brand role manipulation separately with 141 consumers who were randomly assigned to three role manipulations (*M*age = 31.5, *SD* = 2.40, 42% female). We measured the participants measured master perceptions (“The brand looks like a master to me” and “I would obey the brand,” *r* = 0.83), mentor perceptions (“The brand looks like a mentor to me” and “The brand would help me,” *r* = 0.82) and servant perceptions (“The brand looks like a servant to me” and “The brand would obey me,” *r* = 0.84), anchored by 1 = strongly disagree and 7 = strongly agree. As expected, the master manipulation resulted in stronger master perceptions (*M*master = 5.37 vs. *M*mentor = 3.41, *M*servant = 3.28), and the mentor manipulation resulted in stronger mentor perceptions (*M*mentor = 5.11 vs. *M*master = 3.33, *M*servant = 3.42). Additionally, servant manipulation resulted in stronger servant perceptions (*M*servant = 5.12 vs. *M*master = 3.17, *M*mentor = 3.68).

### Measure

First, we tested self-construal manipulation valance similar to study 1. Next, the participants then indicated their attitude toward the advertised brand on a four-item, seven-point scale anchored by ‘bad/good,’ ‘unfavorable/favorable,’ ‘negative/positive,’ and ‘unappealing/appealing’ (Jeong, 2008; Chang et al., 2017). Finally, the participants completed demographic questions (i.e., gender and age).

### Results

#### Manipulation Check

We also invited two independent researchers to code and judge each statement as either interdependent or independent, similar to Experiment 1 and 2. As a result, participants in the independent prime wrote more individualistic statements than those in the interdependent prime (*M* = 5.61 vs. 4.39, *SD* = 1.32 vs. 1.31, β = 5.23, *t* = 11.26, *p* < 0.01), whereas participants in the interdependent prime wrote more collectivistic statements than those in the independent prime (*M* = 5.56 vs. 4.38, *SD* = 1.12 vs. 1.14, β = 2.24, *t* = -5.86, *p* < 0.01).

#### Attitude Toward Advertised Brand

A 3 (anthropomorphized role: master, mentor, and servant) ^∗^ 2 (self-construal: independent, interdependent) analysis of variance (ANOVA) on brand attitude indicated that the interaction effect between anthropomorphism role and self-construal was significant [*F*(2,245) = 17.07, *p* < 0.01, η^2^ = 0.15] (see Table 5).

**Table 5:** Results of two-way analysis of variance for brand attitude.

	*SS*	*df*	*MS*	*F*	*p*	Cohen’ *f*	*Achieved power*
Anthropomorphism role (A)	0.32	2	0.16	0.13	0.88	0.03	0.06
Self-construal (B)	21.77	1	21.76	17.06	0.00	0.25	0.97
A^∗^B	34.14	2	17.07	13.37	0.00	0.31	0.99
Error	312.73	245	1.28				

*^∗^*p*-value ≤ 0.05; ^∗∗^*p*-value ≤ 0.01; and ^∗∗∗^*p*-value ≤ 0.001.*

**FIGURE 3:**
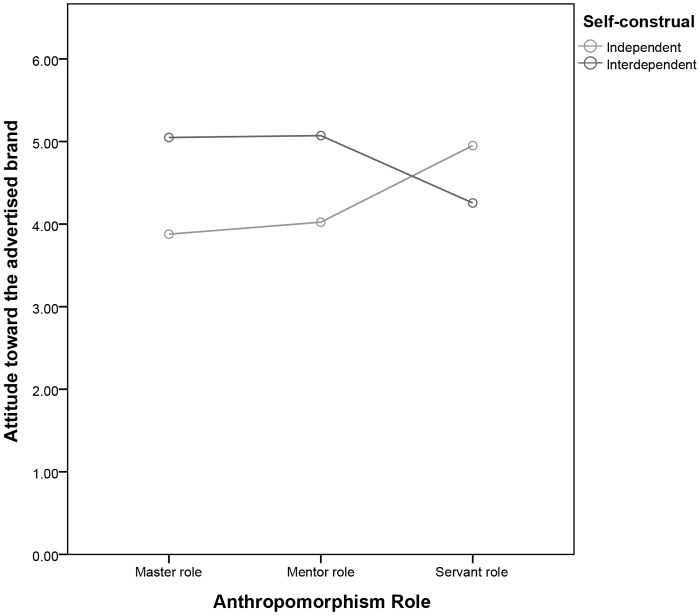
Attitude toward the advertised brand as a function of the anthropomorphism role and self-construal.

We then undertook a *post hoc* analysis. In the master role condition, interdependent consumers responded with more affection than did independent consumers [*M*ind = 3.87 vs. *M*int = 5.04, *SD* = 1.51 vs. 1.39; *F*(1,245) = 22.19, *P* < 0.01]. In addition, interdependent consumers responded with more affection than did independent consumers in the mentor role condition [*M*ind = 4.02 vs. *M*int = 5.07, *SD* = 0.98 vs. 0.89; *F*(1,245) = 18.44, *P* < 0.01]. However, independent consumers responded with more affection than did interdependent consumers in the servant role condition [*M*ind = 4.95 vs. *M*int = 4.26, *SD* = 1.02 vs. 1.19; *F*(1,245) = 8.02, *P* < 0.01] (see Figure 3 and Table 6). The result demonstrate that proposed interaction effect is not affected by product selection. Additionally, we can eliminate the influence of process sequence.

**Table 6:** Post-test between Anthropomorphism role (AR) and Brand Attitude (BA).

AR\BA	Mean and SD	*F*	*P*
Master role frame	*M*ind = 3.87, *SD* = 1.51 vs. *M*int = 5.04, *SD* = 1.39	22.19	^∗∗∗^
Mentor role frame	*M*ind = 4.02, *SD* = 0.98 vs. *M*int = 5.07 *SD* = 0.89	18.44	^∗∗∗^
Servant role frame	*M*ind = 4.95, *SD* = 1.02 vs. *M*int = 4.26, *SD* = 1.19	8.02	^∗∗∗^

*^∗^*p*-value ≤ 0.05; ^∗∗^*p*-value ≤ 0.01; and ^∗∗∗^*p*-value ≤ 0.001.*

## Experiment 4

This experiment serves for multiple purposes. Participants in the prior experiments are only given text messages of anthropomorphism brand, but no visual messages. Guthrie (1993) suggests the forms of anthropomorphism include partial and the literal. Partial anthropomorphizing occurs when people see objects and events as having some important human traits but do not consider the entity as a whole to be human. Thus, this experiment explore the interactive effect between anthropomorphism role and consumer self-construal when they are given the visual messages in this experiment. Besides, the participants in the previous three experiments all are graduate students. To verify the applicability of the experiment, this study was completed online and hosted on the WJC system ^1^. Since WJX launched in 2006, more than 27.97 million questionnaires have been issued by users, and over 1.86 billion questionnaires have been collected. But we exclude the users of WJX under 18 years old, most of whom are financially dependent on their parents and are influenced by others when making purchase decisions.

### Participants

A total of 227 (Mage = 23.5, *SD* = 3.56, 58% female) participation in this study. All subjects provided written informed consent in accordance with the Declaration of Helsinki, similar to foregoing experiments.

### Experimental Design and Procedure

The experiment involved two manipulated factors (anthropomorphism role: master, mentor vs. servant; self-construal: independent vs. interdependent). First, we manipulated the self-construal prompt similar to that of Trafimow et al. (1991). In the independent prime, the participants were told to “Please take 5 min to think and write down how you are different from your family and friends.” In the interdependent prime, the participants were told to “Please take 5 min to think and write down how you are similar to your family and friends.”

Next, we manipulated the anthropomorphism role. The participants were invited to look through an advertisement photo and evaluate a computer brand that want to launch a new computer. We chose a computer as our experimental target because it is a universal smart product of modern life.

The participants in the master condition saw the computer advertisement with master avatar (Appendix D-1), the mentor condition saw the computer advertisement with mentor avatar (Appendix D-2). And the participants in the servant condition saw the computer advertisement with servant avatar (Appendix D-3). We pretest the valence of anthropomorphic role in picture with 45 participants. Participants randomly assigned to three role (master, mentor, servant) advertisement conditions. They were told to evaluate the role type of avatar (“I think the avatar in picture looks like a master,” “I think the avatar in picture looks like a mentor” and “I think the computer avatar in picture looks like a servant,” anchored by 1 = strongly disagree and 7 = strongly agree). The results supported the idea that the manipulation was effective, as the master role situation resulted in stronger master perceptions (*M*master = 5.86 vs. *M*mentor = 3.56, *M*servant = 3.27). The mentor role situation resulted in stronger mentor perceptions (*M*mentor = 5.57 vs. *M*master = 3.70, *M*servant = 3.43). Meanwhile, the mentor role situation resulted in stronger mentor perceptions (*M*servant = 5.93 vs. *M*master = 2.73, *M*mentor = 3.33).

### Measure

Firstly, we tested the manipulation (anthropomorphic branding and self-construal) valance. To test the role of anthropomorphism in manipulation, the participants’ reactions were assessed in terms of whether they considered the branding of the appliance to reflect master leadership (“The brand looks like a master to me” and “I would obey the brand,” *r* = 0.86), mentor leadership (“The brand looks like a mentor to me” and “The brand would help me,” *r* = 0.81) and subservience (“The brand looks like a servant to me” and “The brand would obey me,” *r* = 0.84), anchored by 1 = strongly disagree and 7 = strongly agree. To test their self-construal, the participants were instructed to complete 10 statements beginning with “I am __.” after manipulation (Trafimow et al., 1991; Brewer and Gardner, 1996; Gardner et al., 1999).

Next, the participants then indicated their attitude toward the advertised brand on a four-item, seven-point scale anchored by ‘bad/good,’ ‘unfavorable/favorable,’ ‘negative/positive,’ and ‘unappealing/appealing’ (Jeong, 2008; Chang et al., 2017). Finally, the participants completed demographic questions (i.e., gender and age).

### Results

#### Manipulation Check

As expected, the leader manipulation resulted in stronger leader perceptions (*M*master = 5.28 vs. *M*mentor = 3.57, *M*servant = 3.68), the mentor manipulation in stronger mentor perceptions (*M*master = 3.60 vs. *M*mentor = 5.59, *M*servant = 3.36) and the servant manipulation in stronger servant perceptions (*M*master = 3.09 vs. *M*mentor = 3.21, *M*servant = 5.11).

We also invited two independent researchers to code and judge each statement as either interdependent or independent, similar to foregoing experiments. As a result, participants in the independent prime wrote more individualistic statements than those in the interdependent prime (*M* = 5.54 vs. 4.41, *SD* = 1.26 vs. 1.19, β = 1.13, *t* = 5.93, *p* < 0.01), whereas participants in the interdependent prime wrote more collectivistic statements than those in the independent prime (*M* = 4.07 vs. 5.54, *SD* = 1.26 vs. 1.12, β = -1.47, *t* = -8.84, *p* < 0.01).

#### Attitude Toward Advertised Brand

A 3 (anthropomorphized role: master, mentor and servant) ^∗^ 2 (self-construal: independent, interdependent) analysis of variance (ANOVA) on brand attitude indicated that the interaction effect between anthropomorphism role and self-construal was significant [*F*(2,221) = 22.51, *p* < 0.01, η^2^ = 0.16] (see Table 7).

**Table 7:** Results of two-way analysis of variance for brand attitude.

	*SS*	*df*	*MS*	*F*	*p*	Cohen’ *f*	*Achieved power*
Anthropomorphism role (A)	3.17	2	1.58	1.22	0.29	0.10	0.17
Self-construal (B)	9.76	1	9.76	7.53	0.00	0.18	0.49
A^∗^B	45.02	2	22.51	17.38	0.00	0.43	0.99
Error	286.24	221	1.29				

*^∗^*p*-value ≤ 0.05; ^∗∗^*p*-value ≤ 0.01; and ^∗∗∗^*p*-value ≤ 0.001.*

**FIGURE 4:**
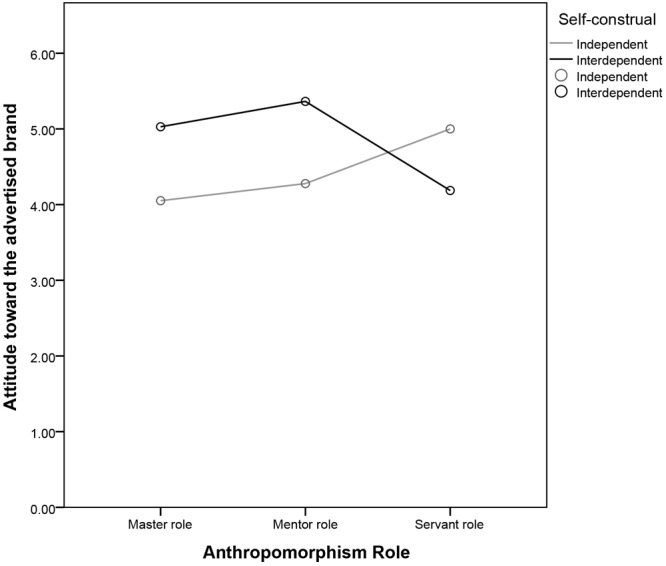
Attitude toward the advertised brand as a function of the anthropomorphism role and self-construal.

We then undertook a *post hoc* analysis. In the master role condition, interdependent consumers responded with more affection than did independent consumers [*M*ind = 4.05 vs. *M*int = 5.03, *SD* = 1.15 vs. 1.01; *F*(1,221) = 13.60, *P* < 0.01]. In addition, interdependent consumers responded with more affection than did independent consumers in the mentor role condition [*M*ind = 4.28 vs. *M*int = 5.36, *SD* = 1.09 vs. 1.34; *F*(1,221) = 15.67, *P* < 0.01]. However, independent consumers responded with more affection than did interdependent consumers in the servant role condition [*M*ind = 5.00 vs. *M*int = 4.19, *SD* = 1.02 vs. 1.21; *F*(1,221) = 10.73, *P* < 0.01] (see Figure 4 and Table 8). The result demonstrate that the interactive effect between anthropomorphism role and consumer self-construal similar to experiment 1 when they are given the visual messages.

**Table 8:** Post-test between Anthropomorphism role (AR) and Brand Attitude (BA).

AR\BA	Mean and SD	*F*	*P*
Master role frame	*M*ind = 4.05, *SD* = 1.15 vs. *M*int = 5.03, *SD* = 1.01	13.60	^∗∗∗^
Mentor role frame	*M*ind = 4.28, *SD* = 1.09 vs. *M*inter = 5.36, *SD* = 1.34	15.67	^∗∗∗^
Servant role frame	*M*ind = 5.00, *SD* = 1.02 vs. *M*inter = 4.19, *SD* = 1.21	10.73	^∗∗∗^

*^∗^*p*-value ≤ 0.05; ^∗∗^*p*-value ≤ 0.01; and ^∗∗∗^*p*-value ≤ 0.001.*

## General Discussion

The research presented here is based on an investigation of the interactive effects of self-construals and anthropomorphized branding. It provides new insights by demonstrating how many consumers favor brands constructing themselves as superior – whether this is through suggesting they can offer mastery or mentoring. Thus it indicates that consumers do not always favor brands which they feel represent subordination or offer a partnership, despite the extensive literature focusing on the notions of ‘brand-as-partner’ and ‘brand-as-servant’ (Fournier, 1998; Fournier and Alvarez, 2012; Kim and Kramer, 2015). The results of our study suggest that a brand positioning themselves as superior will attract individuals with an interdependent self-construal, thus suggesting ‘The brand is king’ rather than ‘The customer is king.’

Experiment 1 provides evidence of the validity of our central hypotheses that (1) individuals with an interdependent self-construal respond more favorably to an anthropomorphized brand playing superior (master and mentor) roles than toward those acting out subservient roles, and (2) individuals with an interdependent self-construal respond more favorably to such superior (master and mentor) anthropomorphized brands than individuals with an independent self-construal. Additionally, the research distinguishes between two superior roles, master and mentor, based on expressions of behaviors and communications, reflecting the different psychological reasons for followership. An anthropomorphic master brand appears to drive blind followership, a mentor brand to drive rational followership among interdependent consumers, in turn mediating the interactive effects between anthropomorphism and self-construals. We verified the same interaction effect whether the subjects received the text or the visual anthropomorphic information combined with Experiment 4.

Existing literature (Aaker, 1997; Aggarwal and McGill, 2007; Donnolley, 2012; Kim and Kramer, 2015) only considers the consumer and brand relationship. This research tests anthropomorphic strategies when a third party (a gift-recipient) is involved in the relationship between a consumer and a brand. Experiment 2 shows that the giver-recipient relationship moderates the relationship between an anthropomorphized brand and self-construals. Givers prefer the anthropomorphic role that can best meet their needs, as well as those of the gift recipient based on their relationship to them. When gift-givers with an interdependent self-construal perceive recipients as subordinates (superior) in their relationships, they respond with more affection to a brand using master/mentor (servant) anthropomorphism. Additionally, Experiment 3 demonstrated that production selection and experiment process sequence will not affect the proposed effect, which further verified the validity of the result in Experiments 1 and 2.

### Theoretical Contributions

We offer several contributions to the literature with this research. Firstly, our results add to the literature on self-construals and brand connections. Previous Studies have demonstrated that individual self-construals (independent or interdependent) affect advertising appeals (Agrawal and Maheswaran, 2005); country-of-origin brand evaluations (Swaminathan et al., 2007); perceived brand meanings (Escalas and Bettman, 2005) and responses to brand failures (Cheng et al., 2012). However, there is a paucity of research considering anthropomorphic brand strategies, although authors have speculated about self-construals for the past two decades (Triandis, 1989; Singelis, 1994). Additionally, the finding that the involvement of an anthropomorphic brand more closely reflects actual social relationships when we investigate the relationship between brands and self-construals is a new one. Most importantly, this research furthers the understanding of how superior anthropomorphic roles impact consumer psychology, which adds to the literature on consumer-brand psychology. The positive effect of the master role is based on blind followership; in contrast, the mentor role is based on rational followership.

Additionally, these findings demonstrate a new perspective regarding the importance of consumer-brand relationships in anthropomorphic branding. Prior research on anthropomorphism has only identified two important and distinct anthropomorphic roles: servant and partner (Fournier, 1998; Aggarwal and McGill, 2007; Fournier and Alvarez, 2012; Kim and Kramer, 2015). We bring a new role, ‘the brand as superior,’ to extend knowledge of meaningful consumer-brand relationships.

### Practical Contributions

Marketers often use anthropomorphic strategies to engage, resonate with and attract consumers (Yuan and Dennis, 2017). However, the most common anthropomorphic traits suggesting the brand as a partner or a subordinate seem unable to fully satisfy consumers’ psychological needs for the social connections which they have, or for which they seek in their daily lives. Thus, based on the self-construal theory, we explore a new role of superiority in anthropomorphic advertising to compensate for this shortcoming. Current research suggests that enlightened marketers and advertisers consider followers’ traits when formulating anthropomorphic tactics for their brand. When their target consumers are interdependent groups such as young students or individuals with handicaps, who need support, the superior anthropomorphism strategy is more likely to work than that of the servant. However, when their target consumers are independent groups such as the very rich or leaders who embody power, the servant anthropomorphism strategy is likely to serve these consumers better. In addition, from this research, marketers can also understand the mechanisms of consumer psychology and the importance of knowing consumer traits, which will help them to apply congruent anthropomorphic strategies for targeting consumers.

### Limitations and Future Research

We recognize several limitations to this work and propose future research in light of these. Firstly, we only discuss two (interdependent and independent) self-construals in terms of brand connection. Research shows that most individuals have self-concepts comprising both interdependent and independent traits (Triandis, 1989; Agrawal and Maheswaran, 2005). Thus, we hope to be able to extend this study considering dynamic self-construal factor. Moreover, our mediator and dependent variables were measured simultaneously rather than over time, and therefore equivalent models would exist with the paths going in the opposite direction; simply testing models that reverse the paths will not be sufficient to rule out these alternative models (Thoemmes, 2015; Lemmer and Gollwitzer, 2017). Thus, we would measure these two variables over time in further research. In addition to self-construals, we are interested in studying the effects of charismatic leadership, which strongly affects followers’ self-concepts in the interest of the mission articulated by the leader (Shamir et al., 1993). Additionally, although we explore the master, mentor and servant roles, these roles do not represent the complexity of social relationships nor cover the gamut of the types of anthropomorphized characters to represent a brand. For example, Subaru plays the role of the guardian of family safety in its series of advertisements: this role differs from that of servant, mentor and master, in that a guardian has the power and obligation to watch over someone (Wright and Snell, 2005). There is no shortage of research opportunities to investigate the other roles that brands may assume and to explore how those roles may influence consumers, and we hope to be able to do this. Furthermore, the brands described in experiments are judged only by these messages given in the study and not others that brands take, for example, from the manufacture country. Lin and Chen (2017) proved that manufacture country of brand influence the consumer perception. Thus, we take these more brand relevant information into account in further research.

## Ethics Statement

This study was carried out in accordance with the recommendations of the National Central University Research Committee with written informed consent obtained from all subjects. Also, this study was reviewed and approved by the Ethics Committee of the National Central University. All subjects gave written informed consent in accordance with the Declaration of Helsinki.

## Author Contributions

C-HL contributed to the improvement of the initial idea. YH designed the study and collected the data. Both authors conducted the analysis, worked on the first draft and revised the conceptualizations and the final manuscript. Both authors approved the final version of the manuscript.

## Conflict of Interest Statement

The authors declare that the research was conducted in the absence of any commercial or financial relationships that could be construed as a potential conflict of interest. The reviewer ML-P and handling Editor declared their shared affiliation.

## Footnotes

1 www.wjx.cn
